# Evaluation of ChatGPT 4.0 in Thoracic Imaging and Diagnostics

**DOI:** 10.7759/cureus.73741

**Published:** 2024-11-15

**Authors:** Golnaz Lotfian, Keyur Parekh, Mohammed Abdul Sami, Pokhraj P Suthar

**Affiliations:** 1 Department of Diagnostic Radiology and Nuclear Medicine, Rush University Medical Center, Chicago, USA

**Keywords:** ai, gpt, machine learning, natural language processing, nlp, thoracic imaging

## Abstract

Recent advancements in natural language processing (NLP) have profoundly transformed the medical industry, enhancing large cohort data analysis, improving diagnostic capabilities, and streamlining clinical workflows. Among the leading tools in this domain is ChatGPT 4.0 (OpenAI, San Francisco, California, US), a commercial NLP model widely used across various applications. This study evaluates the diagnostic performance of ChatGPT 4.0 specifically in thoracic imaging by assessing its ability to answer diagnostic questions related to this field. We utilized the model to respond to multiple-choice questions derived from thoracic imaging scenarios, followed by rigorous statistical analysis to assess its accuracy and variability across different subgroups.

Our analysis revealed significant variability across different subgroups. Overall, the model achieved an impressive accuracy of 84.9% in diagnosing thoracic radiology questions. It excelled in terminology and diagnostic signs, achieving perfect scores, and demonstrated strong performance in the intensive care and normal anatomy categories, with accuracies of 90% and 80%, respectively.

In pathology subgroups, ChatGPT achieved an average accuracy of 89.1%, particularly excelling in diagnosing infectious pneumonia and atelectasis, though it scored lower in diffuse alveolar disease (66.7%). For disease-related questions, the mean accuracy was 79.1%, with perfect scores in several specific subcategories. However, accuracy was notably lower for vascular disease (50%) and lung cancer (66.7%).

In conclusion, while ChatGPT 4.0 shows strong potential in diagnosing thoracic conditions, the variability identified underscores the necessity for ongoing research and refinement of its transformer architecture. This will enhance its reliability and applicability in broader clinical and patient care settings.

## Introduction

Natural language processing (NLP) advancement and its integration with machine learning (ML) algorithms have far surpassed the rudimentary rule-based systems used for translating languages in the mid-20th century [1,2]. Recent developments, particularly in transformer architecture like generative pre-trained transformers (GPT; OpenAI, San Francisco, California, US) have opened numerous commercial possibilities for artificial intelligence (AI) [2]. These advancements include improved search engine capabilities for research, enhanced voice-to-text functionalities for optimized notetaking and report writing, and sophisticated radiology image interpretation for detecting abnormalities [3]. While NLP and ML integration continues to make significant strides in the medical industry, robust data validation is crucial to test the effectiveness and ensure the trusted use of these pre-trained models in such a critical field [4,5]. In this analysis, we provide a detailed assessment of GPT 4.0’s capability in analyzing and responding to a wide range of board-style questions in the field of thoracic imaging.

## Materials and methods

This study aimed to assess the accuracy of ChatGPT 4.0 (4.0-Turbo) in answering diagnostic questions derived from Thoracic Imaging: A Core Review by Hobbs et al. [6]. We selected 126 random multiple-choice questions from the 540 diagnostic questions in the textbook. The evaluation focused on key subgroups within thoracic imaging to analyze the model’s performance and variability. These subgroups were designed to assess differences in accuracy between questions related to fundamental knowledge and those concerning specific diseases and physiological aspects of thoracic imaging.

**Figure 1:**
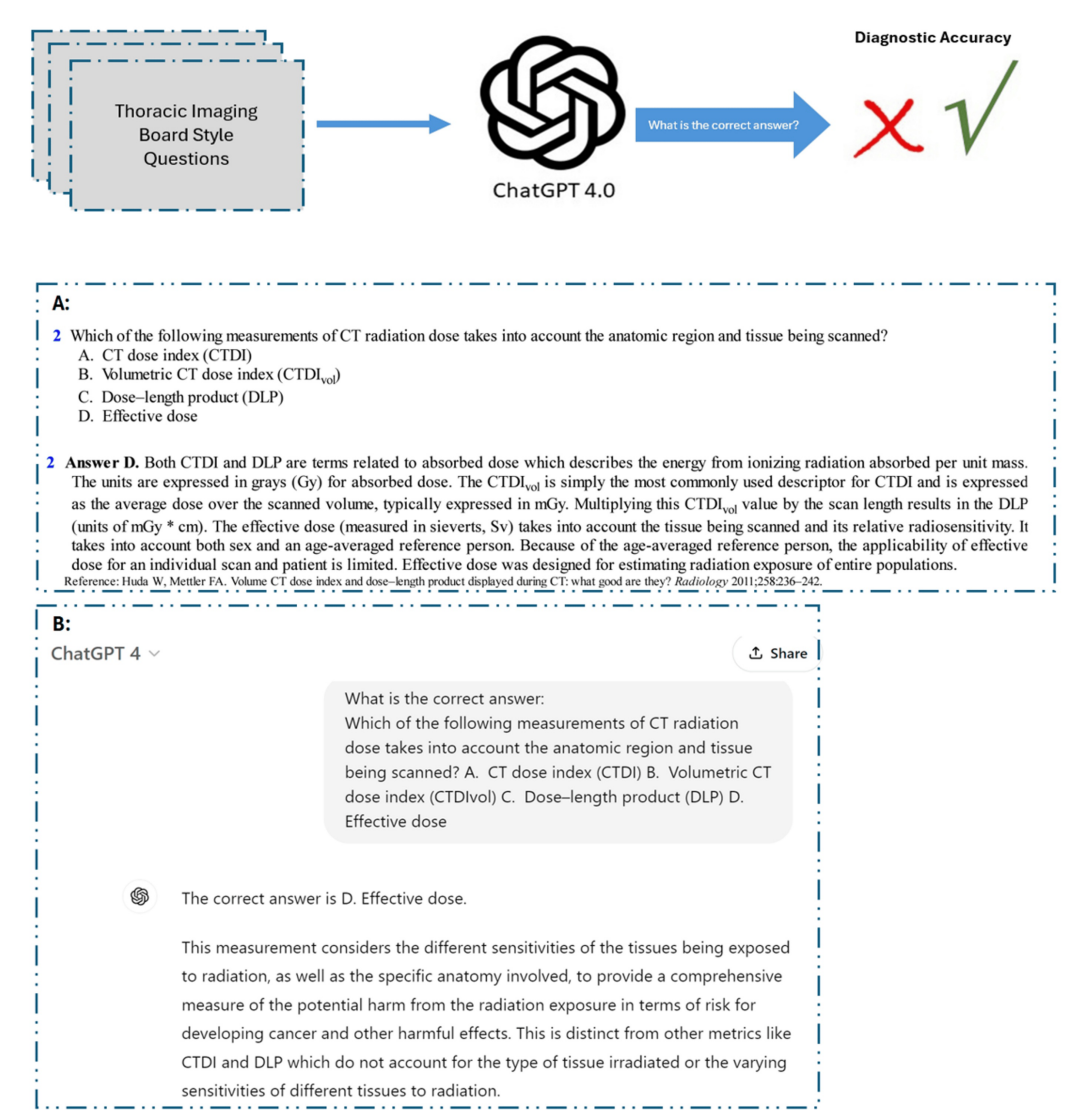
Illustration of provided text question prompt and associated ChatGPT response A: The questions and their associated answers were sourced from the textbook Thoracic Imaging: A Core Review by Hobbs et al. [6], encompassing various subgroups within the field of thoracic imaging; B: ChatGPT 4.0 was presented with each text question along with four potential answer options. The model's responses were recorded for subsequent statistical analysis.

For each question, we provided ChatGPT with a text prompt and four potential choices (a, b, c, and d), querying the model with, 'What is the correct answer?'. The responses were meticulously recorded for subsequent statistical analysis (Figure 1). Independent radiologists reviewed and validated the answers generated by ChatGPT, comparing them with the textbook responses to ensure accuracy and reliability.

The statistical analysis was conducted using a Microsoft Excel spreadsheet (Microsoft Corp., Redmond, WA, US), where we calculated the accuracy of the model as a percentage of correct responses out of the total cases examined. This analysis adhered to the STARD (Standards for Reporting of Diagnostic Accuracy Studies) guidelines, maintaining scientific integrity and reliability throughout the assessment.

## Results

**Figure 2:**
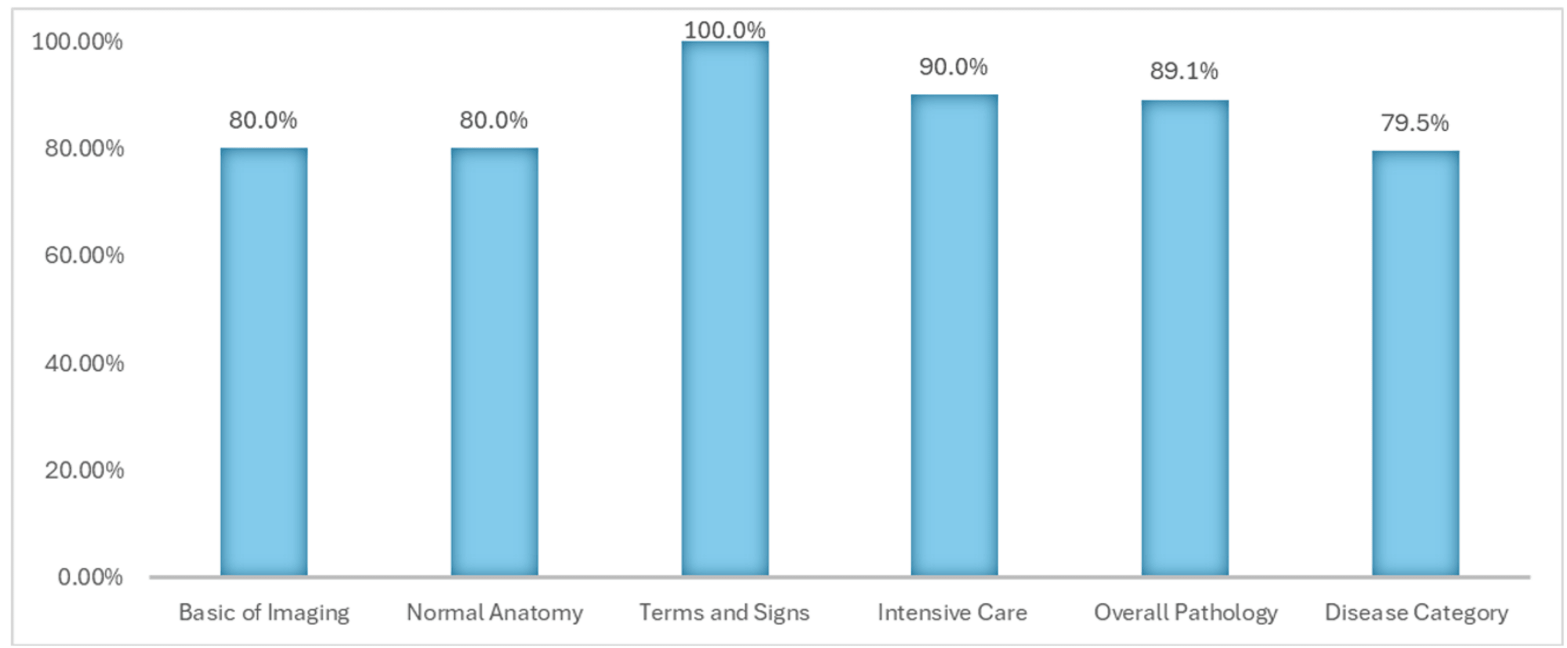
ChatGPT 4.0 capability across six selected subgroups ChatGPT 4.0 achieved an overall accuracy of 84.9% with a standard deviation of 7.46 across 6 selected subgroups. Notably, it demonstrated perfect accuracy of 100% in the 'Terms and Signs' subgroup.

**Figure 3:**
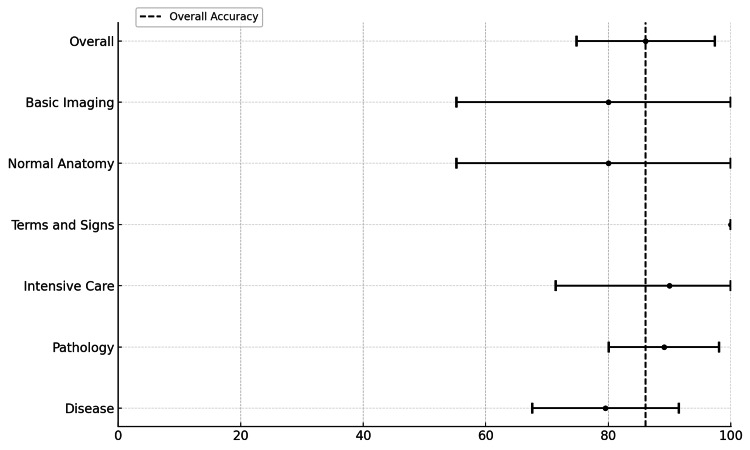
Forest plot across six selected subgroups Forest plot (RR with 95% CI) showing individual subgroup accuracy vs. baseline (overall accuracy).

ChatGPT 4.0 achieved an overall accuracy of 84.9% in responding to the selected assessment questions, with variability observed across six subgroups. The model particularly excelled in questions pertaining to terminology and diagnostic signs, attaining a perfect score of 100%. Additionally, it performed effectively in other categories, correctly answering 80% or more of the questions in intensive care, overall pathology, normal anatomy, and basic imaging (Figures 2, 3).

**Figure 4:**
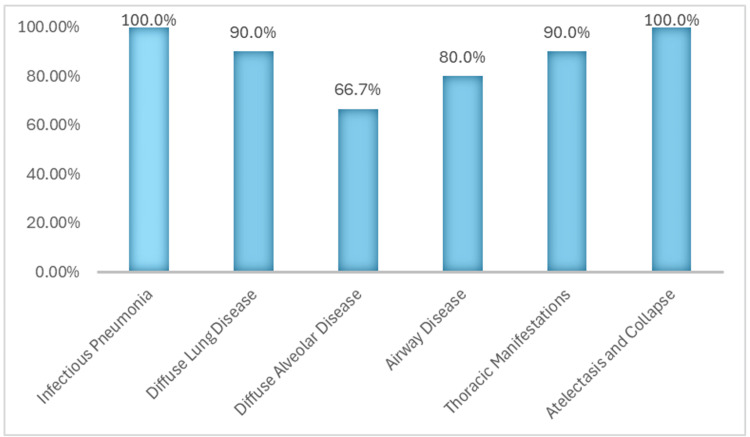
ChatGPT 4.0 capability across the pathology subgroup ChatGPT 4.0 achieved an overall accuracy of 89.1% with a standard deviation of 11.65 in the pathology subgroup. It demonstrated perfect performance, scoring 100% in both the 'Infectious Pneumonia' and 'Atelectasis and Collapse' categories. Additionally, in this subgroup, ChatGPT performed above 80% in several other categories, including pathology-related diseases associated with the airway and thoracic manifestations.

In the pathology subgroup, ChatGPT achieved an average accuracy of 89.1%, with a standard deviation of 11.65. The model demonstrated perfect accuracy, scoring 100% on questions related to infectious pneumonia, atelectasis, and collapse. The lowest accuracy was observed in questions about diffuse alveolar disease, with a mean score of 66.7%. All other subcategories achieved scores of 80% or higher (Figure 4).

**Figure 5:**
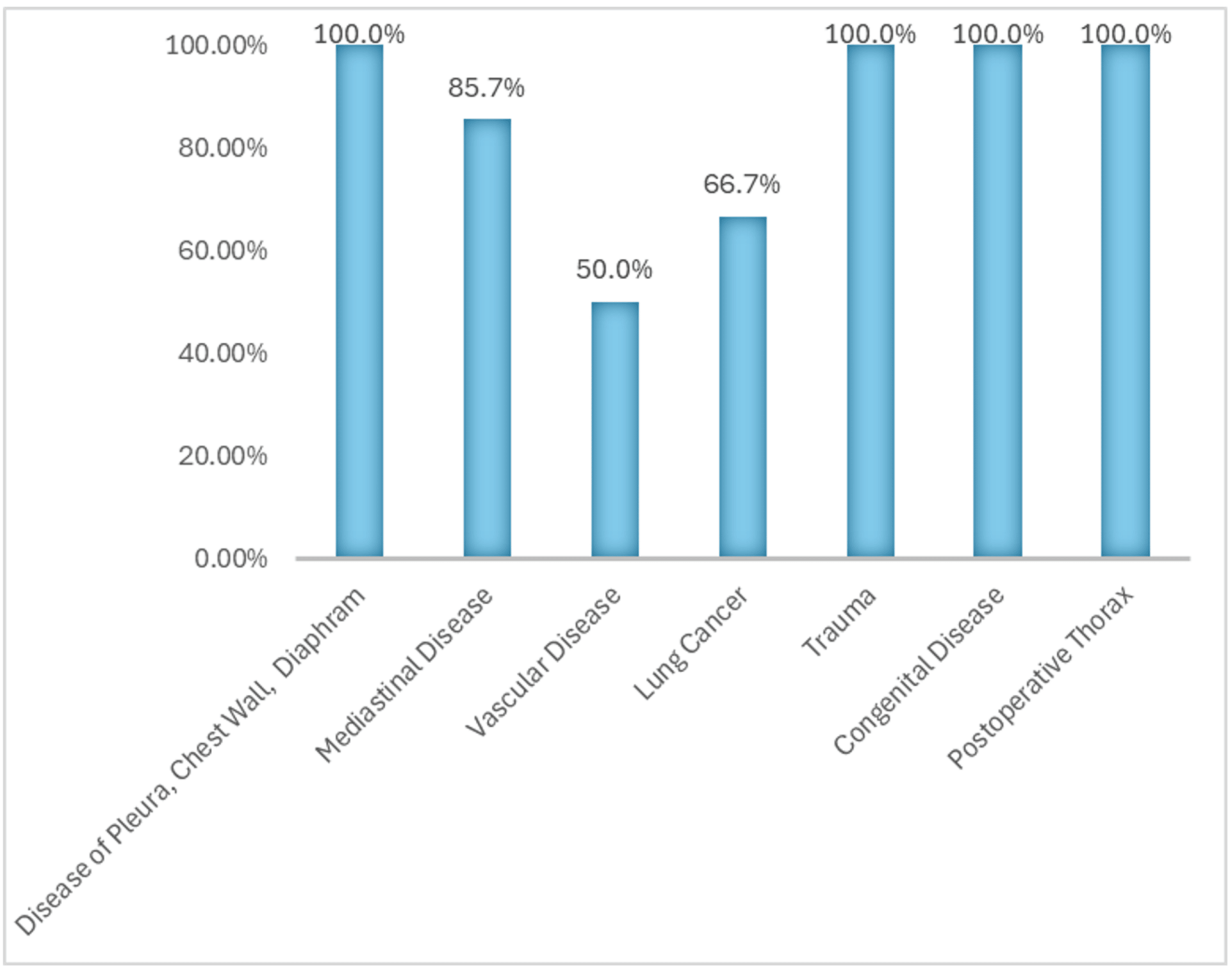
ChatGPT 4.0 capability across the disease subgroup ChatGPT 4.0 achieved an overall accuracy of 79.5% with a standard deviation of 18.72 in the disease subgroup. It demonstrated perfect performance, scoring 100% in multiple categories, including ‘Postoperative Thorax’, ‘Congenital Disease’, and ‘Disease of Pleura, Chest Wall, and Diaphragm’.

ChatGPT 4.0 demonstrated notable reliability in answering targeted disease-related questions (Figure 5). Although the overall mean accuracy for disease-related questions was lower than that for the pathology subcategory, at 79.1%, the model achieved a perfect score of 100% in four specific disease subcategories: pleura-chest wall-diaphragm, trauma, congenital disease, and postoperative thorax. Conversely, the model had the lowest accuracy in the vascular disease subcategory, with a mean score of 50%, and scored 66.7% in lung cancer. Disease-related questions exhibited the highest standard deviation of 18.72, reflecting considerable variability in performance across different disease categories.

**Figure 6:**
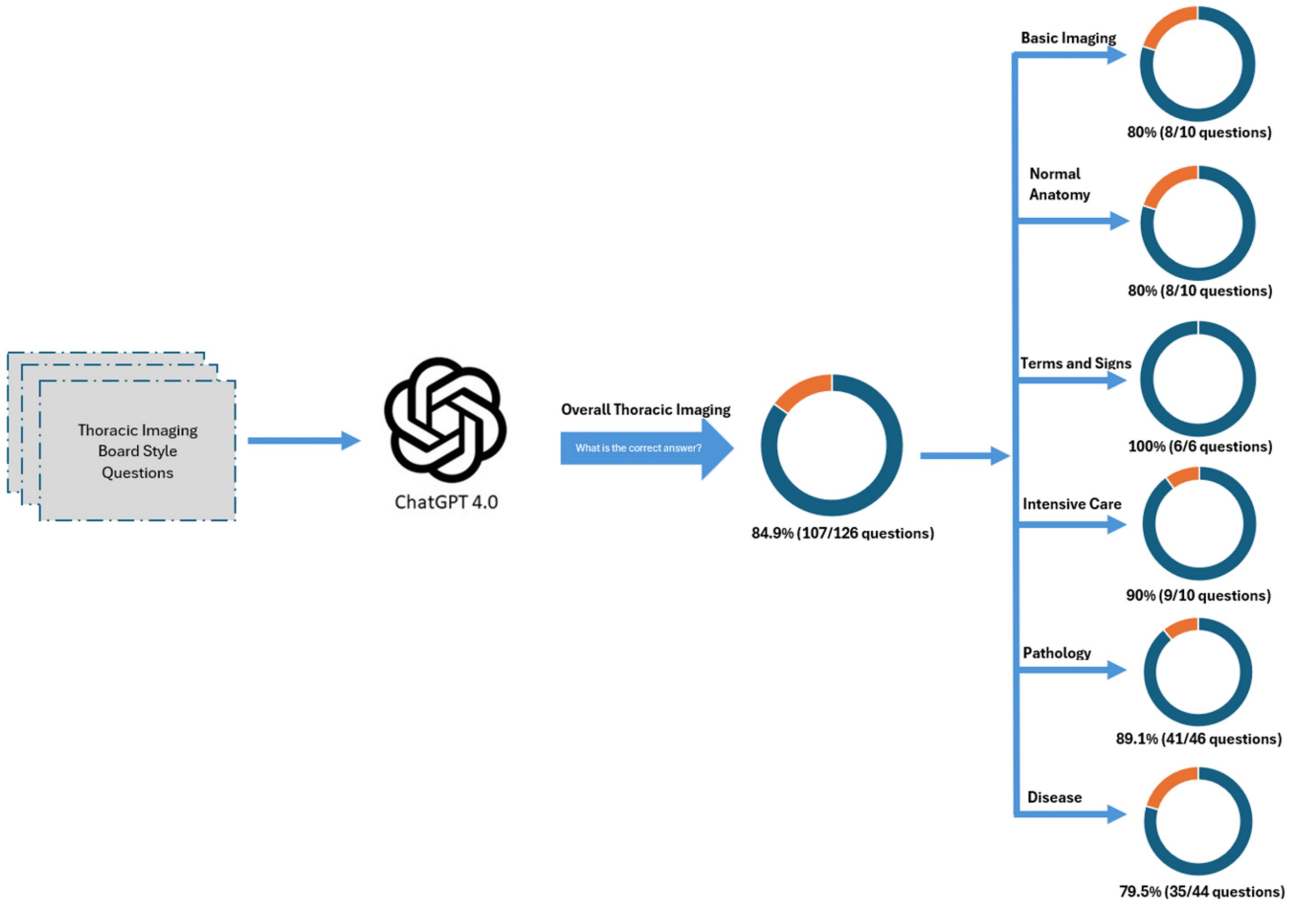
Illustration of diagnostic accuracy of ChatGPT 4.0 in solving thoracic imaging questions across 6 subgroups ChatGPT 4.0 achieved an overall accuracy of 84.9% in responding to thoracic imaging diagnostic board-style questions. The model excelled particularly in the terminology and sign category, where it attained a perfect accuracy of 100%. Additionally, ChatGPT 4.0 demonstrated impressive performance, scoring over 80% accuracy in several subgroups, including pathology, normal anatomy, basic imaging, and intensive care.

In summary, ChatGPT 4.0 demonstrated strong accuracy in answering diagnostic questions across several targeted areas of thoracic imaging, achieving perfect scores in categories such as terminology and diagnostic signs, infectious pneumonia, atelectasis and collapse, chest wall and diaphragm-related diseases, trauma, congenital conditions, and postoperative thoracic diseases (Figure 6). However, the model showed considerable variability in performance in other areas such as vascular disease and lung cancer. This variability underscores the need for continued research and refinement of the transformer architecture used in ChatGPT 4.0 to improve its reliability and applicability in broader clinical and patient care settings.

## Discussion

NLP, with its primary goal of facilitating reliable and accurate interactions between computers and human languages, traces its roots back to the 1950s [1]. The journey began with rudimentary rule-based systems developed for translating languages during the Cold War [1]. These early efforts laid the groundwork for future advancements in the field.

As computational capabilities grew, so did the complexity and sophistication of NLP techniques. The introduction of large datasets, statistical methods, and probabilistic models in the 1980s marked a pivotal shift, leading to the emergence of deep learning and pre-trained models [1-3]. These developments allowed systems to learn from vast amounts of data, enabling them to understand and generate human language with increasing accuracy.

The evolution of NLP reached new heights with the advent of transformer architectures, such as GPT and Bidirectional Encoder Representations from Transformers (BERT; Google AI, Mountain View, CA, US), which have revolutionized language analysis capabilities [2,3]. These models leverage attention mechanisms to understand context, semantics, and nuances in language, leading to significant improvements in tasks ranging from machine translation to sentiment analysis [4].

In recent years, the integration of NLP in specialized fields like radiology has become increasingly prominent [5]. Radiologists face the challenge of interpreting vast amounts of data and reports, often leading to information overload. NLP technologies have the potential to enhance workflow efficiency, improve diagnostic accuracy, and streamline communication within healthcare systems [5,7]. By automating the extraction of relevant information from unstructured text, NLP can assist radiologists in making more informed decisions and providing timely patient care [7].

This study aimed to evaluate ChatGPT 4.0, a widely recognized commercial NLP architecture, focusing on its accuracy and reliability in responding to targeted thoracic imaging clinical questions. By assessing its performance, we aim to explore the potential of advanced NLP models in enhancing diagnostic processes and their implications for the future of radiology.

Integration of NLP and radiology

The integration of NLP and AI technologies in radiology is becoming increasingly evident in daily practice [5]. Commercial voice-to-text solutions, such as PowerScribe (Nuance Communications, Burlington, MA, US), Dragon Medical (Nuance Communications, Burlington, MA, US), and IBM Watson (IBM Corp., Armonk, NY, US), are widely adopted in radiology reading rooms worldwide [8,9]. PowerScribe, in particular, enables radiologists to dictate reports using advanced voice recognition technology, which transcribes their speech in real-time [8]. This not only enhances efficiency in note-taking and report documentation but also allows clinicians to focus more on patient care and less on administrative tasks. By integrating seamlessly with various imaging systems and electronic health records (EHRs), PowerScribe retrieves patient data, images, and prior reports automatically, streamlining the reporting process [8-10]. Additionally, it facilitates the creation of structured reports, improving clarity and consistency in documentation while its natural language processing capabilities help identify key phrases and clinical findings [9]. This comprehensive approach to report generation can significantly reduce turnaround times, enhance communication between radiologists and referring physicians, and reduce report-related error potential [9-11].

Recent advancements in AI, particularly the development of pre-trained transformer models like T5, have further revolutionized the field [12]. These deep-learning platforms can efficiently extract targeted information, identify anomalies, and highlight details of interest from previously generated reports [12]. As a result, they significantly streamline the medical reporting process and facilitate research initiatives. Notably, some studies have reported these pre-trained models achieving accuracy rates of up to 70% in identifying relevant clinical data [12,13].

Despite the impressive strides in machine learning's capacity to contextualize complex clinical data and texts, challenges remain regarding its ability to analyze imaging directly [14,15]. While commercial tools, such as RadAI (RadAI Inc., San Francisco, CA) and Zebra Medical Vision (Zebra Medical Vision Ltd., Ness Ziona, Israel), have demonstrated improving performance in interpreting imaging reports and detecting abnormalities in targeted anatomical structures [16,17], our study indicates that the ability of machine learning to provide accurate insights from contextualized text necessitates rigorous data validation and ongoing refinement. This is crucial for expanding the clinical utility of such tools and ensuring their reliability in real-world applications. Continued research in this domain is vital. As we develop more sophisticated models and accumulate larger datasets, the potential for AI and NLP to enhance image understanding and clinical decision-making grows exponentially. The future of radiology lies in our ability to harness these advancements effectively, ensuring that we can leverage AI-driven tools to improve patient outcomes and streamline workflows in clinical settings.

Study limitations

This study reveals significant variability in ChatGPT 4.0's performance when evaluating thoracic imaging questions and diagnostics. Due to the lack of access to the underlying transformer logic and the inherent biases present in its pre-training, pinpointing the root causes of this variability across different subgroups is challenging. Moreover, we have not analyzed the reproducibility of ChatGPT's responses, which is critical for determining the consistency of its performance over time. Additionally, the small sample sizes within certain subgroups may have influenced the results obtained. Ongoing data validation studies like the one presented here are essential for accurately assessing AI's capabilities and limitations in diagnostic radiology. Such efforts will contribute to a clearer understanding of how these models can be effectively integrated into clinical practice.

## Conclusions

This study underscores the advancements made by ChatGPT 4.0 in conceptualizing and accurately answering diagnostic questions in thoracic imaging. The model exhibited robust performance across multiple categories, achieving perfect scores of 100% in areas such as terminology and diagnostic signs, infectious pneumonia, and atelectasis and collapse, as detailed in the results section. These achievements highlight the model’s potential to significantly contribute to research and data analysis.

Nevertheless, the study also identified notable variability in ChatGPT 4.0’s performance. In some diagnostic categories, particularly those involving disease analysis, the model's accuracy dropped to as low as 50%, accompanied by a high standard deviation of 20%. This variability emphasizes the need for continued refinement to improve the model’s consistency, reliability, and potential use in clinical settings.
